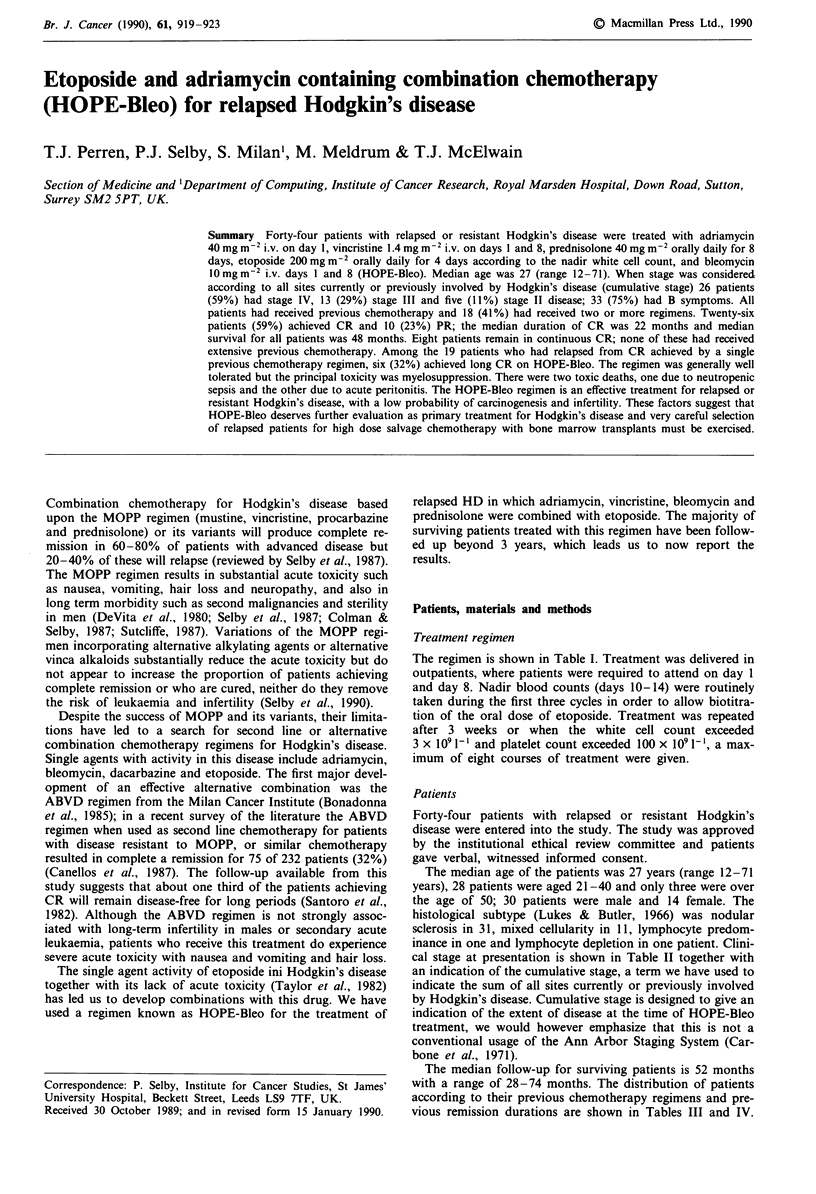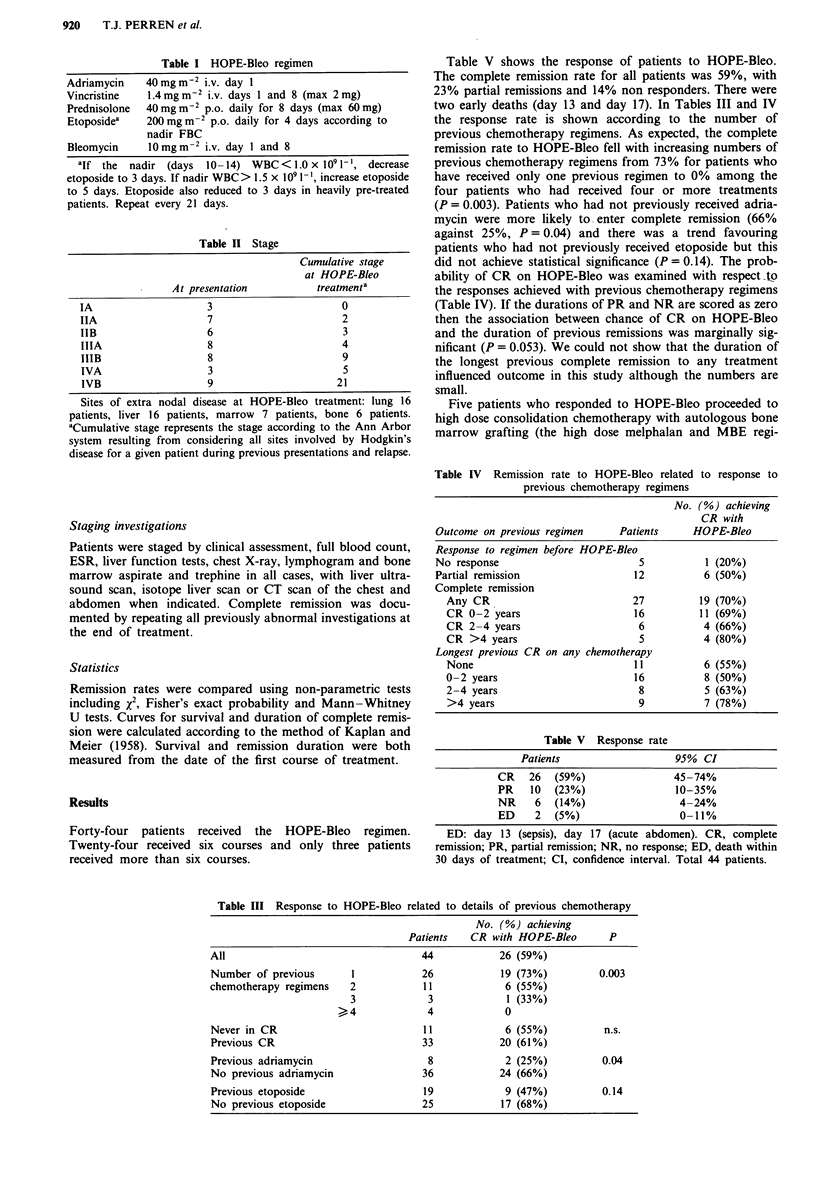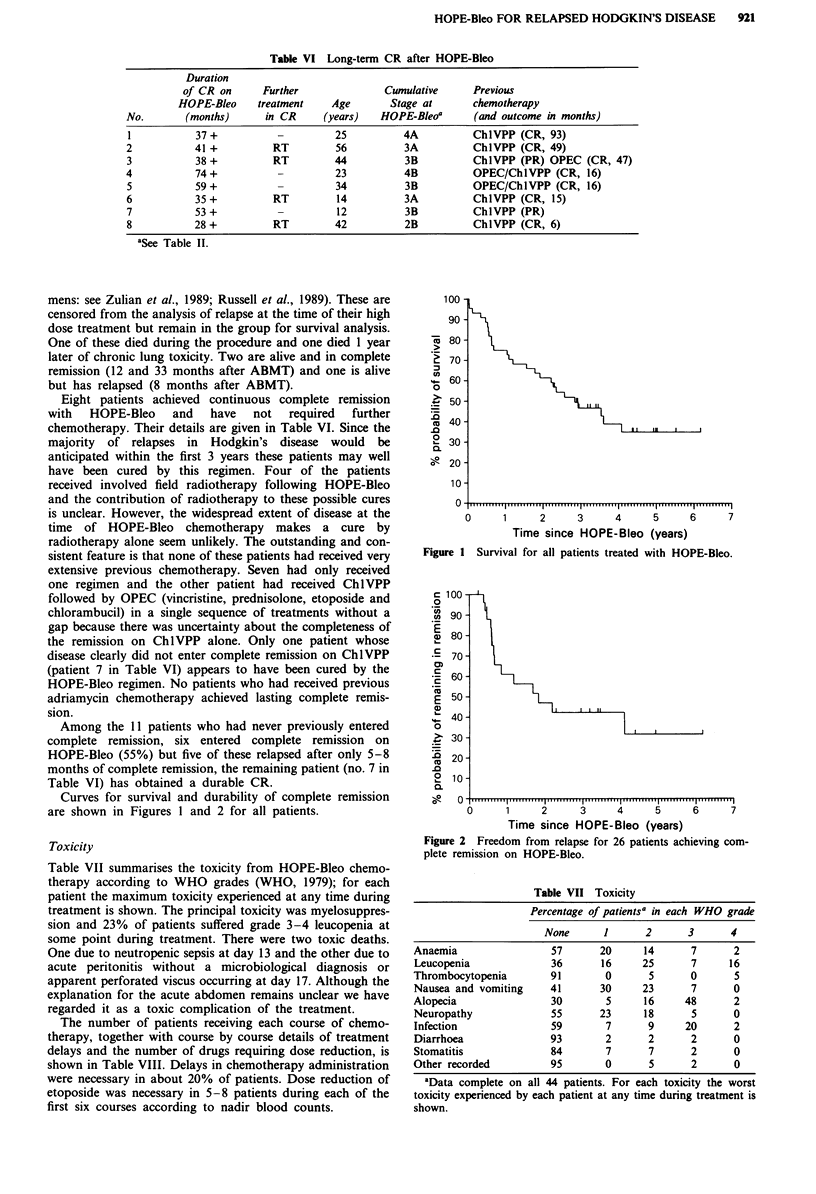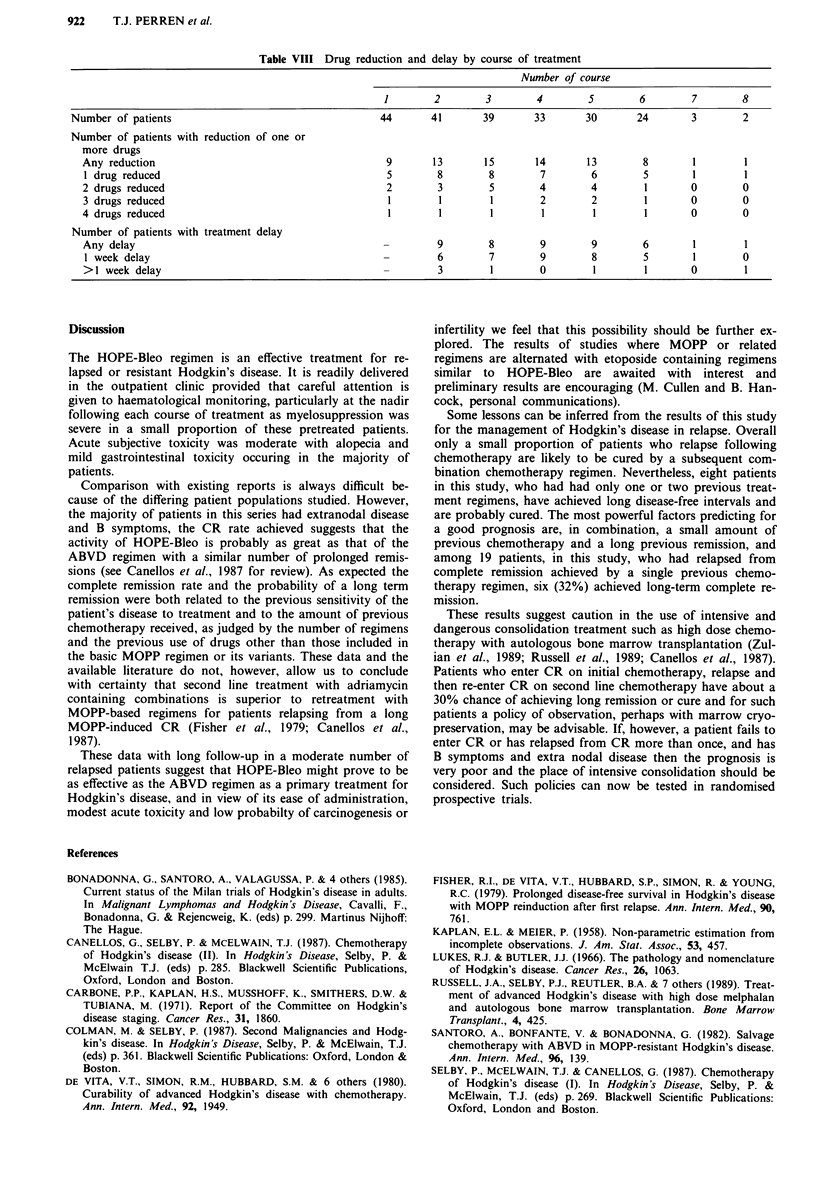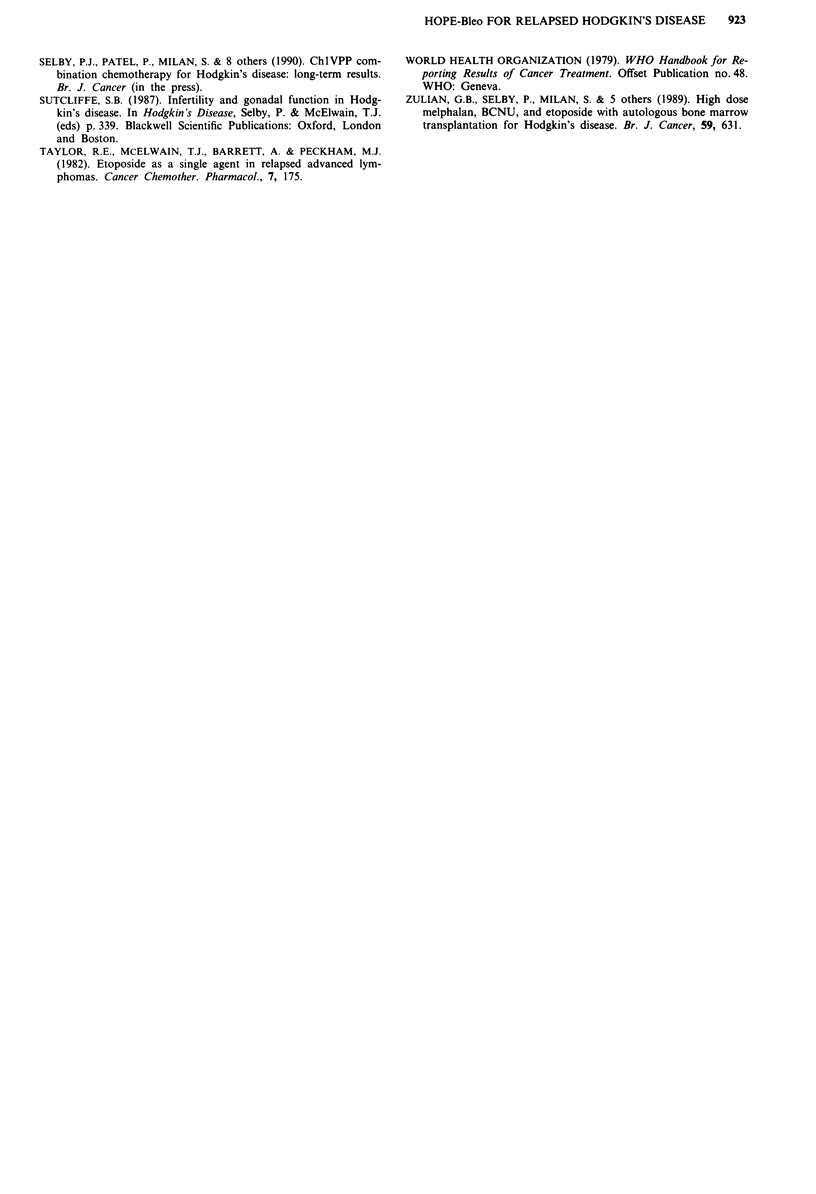# Etoposide and adriamycin containing combination chemotherapy (HOPE-Bleo) for relapsed Hodgkin's disease.

**DOI:** 10.1038/bjc.1990.206

**Published:** 1990-06

**Authors:** T. J. Perren, P. J. Selby, S. Milan, M. Meldrum, T. J. McElwain

**Affiliations:** Section of Medicine, Institute of Cancer Research, Royal Marsden Hospital, Sutton, Surrey, UK.

## Abstract

Forty-four patients with relapsed or resistant Hodgkin's disease were treated with adriamycin 40 mg m-2 i.v. on day 1, vincristine 1.4 mg m-2 i.v. on days 1 and 8, prednisolone 40 mg m-2 orally daily for 8 days, etoposide 200 mg m-2 orally daily for 4 days according to the nadir white cell count, and bleomycin 10 mg m-2 i.v. days 1 and 8 (HOPE-Bleo). Median age was 27 (range 12-71). When stage was considered according to all sites currently or previously involved by Hodgkin's disease (cumulative stage) 26 patients (59%) had stage IV, 13 (29%) stage III and five (11%) stage II disease; 33 (75%) had B symptoms. All patients had received previous chemotherapy and 18 (41%) had received two or more regimens. Twenty-six patients (59%) achieved CR and 10 (23%) PR; the median duration of CR was 22 months and median survival for all patients was 48 months. Eight patients remain in continuous CR; none of these had received extensive previous chemotherapy. Among the 19 patients who had relapsed from CR achieved by a single previous chemotherapy regimen, six (32%) achieved long CR on HOPE-Bleo. The regimen was generally well tolerated but the principal toxicity was myelosuppression. There were two toxic deaths, one due to neutropenic sepsis and the other due to acute peritonitis. The HOPE-Bleo regimen is an effective treatment for relapsed or resistant Hodgkin's disease, with a low probability of carcinogenesis and infertility. These factors suggest that HOPE-Bleo deserves further evaluation as primary treatment for Hodgkin's disease and very careful selection of relapsed patients for high dose salvage chemotherapy with bone marrow transplants must be exercised.


Br. J. Cancer (1990), 61, 919-923 © Macmillan Press Ltd., 1990

### Etoposide and adriamycin containing combination chemotherapy (HOPE-Bleo) for relapsed Hodgkin's disease

T.J. Perren, P.J. Selby, S. Milan[1], M. Meldrum & T.J. McElwain

*Section of Medicine and [1]Department of Computing, Institute of Cancer Research, Royal Marsden Hospital, Down Road, Sutton, Surrey SM2 5PT, UK.*

**Summary** Forty-four patients with relapsed or resistant Hodgkin's disease were treated with adriamycin 40 mg $m^{-2}$ i.v. on day 1, vincristine 1.4 mg $m^{-2}$ i.v. on days 1 and 8, prednisolone 40 mg $m^{-2}$ orally daily for 8 days, etoposide 200 mg $m^{-2}$ orally daily for 4 days according to the nadir white cell count, and bleomycin 10 mg $m^{-2}$ i.v. days 1 and 8 (HOPE-Bleo). Median age was 27 (range 12–71). When stage was considered according to all sites currently or previously involved by Hodgkin's disease (cumulative stage) 26 patients (59%) had stage IV, 13 (29%) stage III and five (11%) stage II disease; 33 (75%) had B symptoms. All patients had received previous chemotherapy and 18 (41%) had received two or more regimens. Twenty-six patients (59%) achieved CR and 10 (23%) PR; the median duration of CR was 22 months and median survival for all patients was 48 months. Eight patients remain in continuous CR; none of these had received extensive previous chemotherapy. Among the 19 patients who had relapsed from CR achieved by a single previous chemotherapy regimen, six (32%) achieved long CR on HOPE-Bleo. The regimen was generally well tolerated but the principal toxicity was myelosuppression. There were two toxic deaths, one due to neutropenic sepsis and the other due to acute peritonitis. The HOPE-Bleo regimen is an effective treatment for relapsed or resistant Hodgkin's disease, with a low probability of carcinogenesis and infertility. These factors suggest that HOPE-Bleo deserves further evaluation as primary treatment for Hodgkin's disease and very careful selection of relapsed patients for high dose salvage chemotherapy with bone marrow transplants must be exercised.

Combination chemotherapy for Hodgkin's disease based upon the MOPP regimen (mustine, vincristine, procarbazine and prednisolone) or its variants will produce complete remission in 60–80% of patients with advanced disease but 20–40% of these will relapse (reviewed by Selby *et al.*, 1987). The MOPP regimen results in substantial acute toxicity such as nausea, vomiting, hair loss and neuropathy, and also in long term morbidity such as second malignancies and sterility in men (DeVita *et al.*, 1980; Selby *et al.*, 1987; Colman & Selby, 1987; Sutcliffe, 1987). Variations of the MOPP regimen incorporating alternative alkylating agents or alternative vinca alkaloids substantially reduce the acute toxicity but do not appear to increase the proportion of patients achieving complete remission or who are cured, neither do they remove the risk of leukaemia and infertility (Selby *et al.*, 1990).

Despite the success of MOPP and its variants, their limitations have led to a search for second line or alternative combination chemotherapy regimens for Hodgkin's disease. Single agents with activity in this disease include adriamycin, bleomycin, dacarbazine and etoposide. The first major development of an effective alternative combination was the ABVD regimen from the Milan Cancer Institute (Bonadonna *et al.*, 1985); in a recent survey of the literature the ABVD regimen when used as second line chemotherapy for patients with disease resistant to MOPP, or similar chemotherapy resulted in complete a remission for 75 of 232 patients (32%) (Canellos *et al.*, 1987). The follow-up available from this study suggests that about one third of the patients achieving CR will remain disease-free for long periods (Santoro *et al.*, 1982). Although the ABVD regimen is not strongly associated with long-term infertility in males or secondary acute leukaemia, patients who receive this treatment do experience severe acute toxicity with nausea and vomiting and hair loss.

The single agent activity of etoposide ini Hodgkin's disease together with its lack of acute toxicity (Taylor *et al.*, 1982) has led us to develop combinations with this drug. We have used a regimen known as HOPE-Bleo for the treatment of relapsed HD in which adriamycin, vincristine, bleomycin and prednisolone were combined with etoposide. The majority of surviving patients treated with this regimen have been followed up beyond 3 years, which leads us to now report the results.

Correspondence: P. Selby, Institute for Cancer Studies, St James' University Hospital, Beckett Street, Leeds LS9 7TF, UK.
Received 30 October 1989; and in revised form 15 January 1990.

### Patients, materials and methods

### *Treatment regimen*

The regimen is shown in Table I. Treatment was delivered in outpatients, where patients were required to attend on day 1 and day 8. Nadir blood counts (days 10–14) were routinely taken during the first three cycles in order to allow biotitration of the oral dose of etoposide. Treatment was repeated after 3 weeks or when the white cell count exceeded $3 \times 10^9 l^{-1}$ and platelet count exceeded $100 \times 10^9 l^{-1}$, a maximum of eight courses of treatment were given.

### *Patients*

Forty-four patients with relapsed or resistant Hodgkin's disease were entered into the study. The study was approved by the institutional ethical review committee and patients gave verbal, witnessed informed consent.

The median age of the patients was 27 years (range 12–71 years), 28 patients were aged 21–40 and only three were over the age of 50; 30 patients were male and 14 female. The histological subtype (Lukes & Butler, 1966) was nodular sclerosis in 31, mixed cellularity in 11, lymphocyte predominance in one and lymphocyte depletion in one patient. Clinical stage at presentation is shown in Table II together with an indication of the cumulative stage, a term we have used to indicate the sum of all sites currently or previously involved by Hodgkin's disease. Cumulative stage is designed to give an indication of the extent of disease at the time of HOPE-Bleo treatment, we would however emphasize that this is not a conventional usage of the Ann Arbor Staging System (Carbone *et al.*, 1971).

The median follow-up for surviving patients is 52 months with a range of 28–74 months. The distribution of patients according to their previous chemotherapy regimens and previous remission durations are shown in Tables III and IV.

**Table I** HOPE-Bleo regimen

| | |
|---|---|
| Adriamycin | $40\,mg\,m^{-2}$ i.v. day 1 |
| Vincristine | $1.4\,mg\,m^{-2}$ i.v. days 1 and 8 (max 2 mg) |
| Prednisolone | $40\,mg\,m^{-2}$ p.o. daily for 8 days (max 60 mg) |
| Etoposide[a] | $200\,mg\,m^{-2}$ p.o. daily for 4 days according to nadir FBC |
| Bleomycin | $10\,mg\,m^{-2}$ i.v. day 1 and 8 |

[a]If the nadir (days 10–14) WBC $<1.0\times10^9\,l^{-1}$, decrease etoposide to 3 days. If nadir WBC $>1.5\times10^9\,l^{-1}$, increase etoposide to 5 days. Etoposide also reduced to 3 days in heavily pre-treated patients. Repeat every 21 days.

**Table II** Stage

| | *At presentation* | *Cumulative stage at HOPE-Bleo treatment*[a] |
|---|---|---|
| IA | 3 | 0 |
| IIA | 7 | 2 |
| IIB | 6 | 3 |
| IIIA | 8 | 4 |
| IIIB | 8 | 9 |
| IVA | 3 | 5 |
| IVB | 9 | 21 |

Sites of extra nodal disease at HOPE-Bleo treatment: lung 16 patients, liver 16 patients, marrow 7 patients, bone 6 patients. [a]Cumulative stage represents the stage according to the Ann Arbor system resulting from considering all sites involved by Hodgkin's disease for a given patient during previous presentations and relapse.

### *Staging investigations*

Patients were staged by clinical assessment, full blood count, ESR, liver function tests, chest X-ray, lymphogram and bone marrow aspirate and trephine in all cases, with liver ultrasound scan, isotope liver scan or CT scan of the chest and abdomen when indicated. Complete remission was documented by repeating all previously abnormal investigations at the end of treatment.

### *Statistics*

Remission rates were compared using non-parametric tests including $\chi^2$, Fisher's exact probability and Mann–Whitney U tests. Curves for survival and duration of complete remission were calculated according to the method of Kaplan and Meier (1958). Survival and remission duration were both measured from the date of the first course of treatment.

### Results

Forty-four patients received the HOPE-Bleo regimen. Twenty-four received six courses and only three patients received more than six courses.

Table V shows the response of patients to HOPE-Bleo. The complete remission rate for all patients was 59%, with 23% partial remissions and 14% non responders. There were two early deaths (day 13 and day 17). In Tables III and IV the response rate is shown according to the number of previous chemotherapy regimens. As expected, the complete remission rate to HOPE-Bleo fell with increasing numbers of previous chemotherapy regimens from 73% for patients who have received only one previous regimen to 0% among the four patients who had received four or more treatments ($P = 0.003$). Patients who had not previously received adriamycin were more likely to enter complete remission (66% against 25%, $P = 0.04$) and there was a trend favouring patients who had not previously received etoposide but this did not achieve statistical significance ($P = 0.14$). The probability of CR on HOPE-Bleo was examined with respect to the responses achieved with previous chemotherapy regimens (Table IV). If the durations of PR and NR are scored as zero then the association between chance of CR on HOPE-Bleo and the duration of previous remissions was marginally significant ($P = 0.053$). We could not show that the duration of the longest previous complete remission to any treatment influenced outcome in this study although the numbers are small.

Five patients who responded to HOPE-Bleo proceeded to high dose consolidation chemotherapy with autologous bone marrow grafting (the high dose melphalan and MBE regi-

**Table IV** Remission rate to HOPE-Bleo related to response to previous chemotherapy regimens

| *Outcome on previous regimen* | *Patients* | *No. (%) achieving CR with HOPE-Bleo* |
|---|---|---|
| *Response to regimen before HOPE-Bleo* | | |
| No response | 5 | 1 (20%) |
| Partial remission | 12 | 6 (50%) |
| Complete remission | | |
| Any CR | 27 | 19 (70%) |
| CR 0–2 years | 16 | 11 (69%) |
| CR 2–4 years | 6 | 4 (66%) |
| CR >4 years | 5 | 4 (80%) |
| *Longest previous CR on any chemotherapy* | | |
| None | 11 | 6 (55%) |
| 0–2 years | 16 | 8 (50%) |
| 2–4 years | 8 | 5 (63%) |
| >4 years | 9 | 7 (78%) |

**Table V** Response rate

| | *Patients* | | *95% CI* |
|---|---|---|---|
| CR | 26 | (59%) | 45–74% |
| PR | 10 | (23%) | 10–35% |
| NR | 6 | (14%) | 4–24% |
| ED | 2 | (5%) | 0–11% |

ED: day 13 (sepsis), day 17 (acute abdomen). CR, complete remission; PR, partial remission; NR, no response; ED, death within 30 days of treatment; CI, confidence interval. Total 44 patients.

**Table III** Response to HOPE-Bleo related to details of previous chemotherapy

| | | *Patients* | *No. (%) achieving CR with HOPE-Bleo* | *P* |
|---|---|---|---|---|
| All | | 44 | 26 (59%) | |
| Number of previous | 1 | 26 | 19 (73%) | 0.003 |
| chemotherapy regimens | 2 | 11 | 6 (55%) | |
| | 3 | 3 | 1 (33%) | |
| | ≥4 | 4 | 0 | |
| Never in CR | | 11 | 6 (55%) | n.s. |
| Previous CR | | 33 | 20 (61%) | |
| Previous adriamycin | | 8 | 2 (25%) | 0.04 |
| No previous adriamycin | | 36 | 24 (66%) | |
| Previous etoposide | | 19 | 9 (47%) | 0.14 |
| No previous etoposide | | 25 | 17 (68%) | |

**Table VI** Long-term CR after HOPE-Bleo

| *No.* | *Duration of CR on HOPE-Bleo (months)* | *Further treatment in CR* | *Age (years)* | *Cumulative Stage at HOPE-Bleo*[a] | *Previous chemotherapy (and outcome in months)* |
|---|---|---|---|---|---|
| 1 | 37 + | – | 25 | 4A | ChlVPP (CR, 93) |
| 2 | 41 + | RT | 56 | 3A | ChlVPP (CR, 49) |
| 3 | 38 + | RT | 44 | 3B | ChlVPP (PR) OPEC (CR, 47) |
| 4 | 74 + | – | 23 | 4B | OPEC/ChlVPP (CR, 16) |
| 5 | 59 + | – | 34 | 3B | OPEC/ChlVPP (CR, 16) |
| 6 | 35 + | RT | 14 | 3A | ChlVPP (CR, 15) |
| 7 | 53 + | – | 12 | 3B | ChlVPP (PR) |
| 8 | 28 + | RT | 42 | 2B | ChlVPP (CR, 6) |

[a]See Table II.

mens: see Zulian *et al.*, 1989; Russell *et al.*, 1989). These are censored from the analysis of relapse at the time of their high dose treatment but remain in the group for survival analysis. One of these died during the procedure and one died 1 year later of chronic lung toxicity. Two are alive and in complete remission (12 and 33 months after ABMT) and one is alive but has relapsed (8 months after ABMT).

Eight patients achieved continuous complete remission with HOPE-Bleo and have not required further chemotherapy. Their details are given in Table VI. Since the majority of relapses in Hodgkin's disease would be anticipated within the first 3 years these patients may well have been cured by this regimen. Four of the patients received involved field radiotherapy following HOPE-Bleo and the contribution of radiotherapy to these possible cures is unclear. However, the widespread extent of disease at the time of HOPE-Bleo chemotherapy makes a cure by radiotherapy alone seem unlikely. The outstanding and consistent feature is that none of these patients had received very extensive previous chemotherapy. Seven had only received one regimen and the other patient had received ChlVPP followed by OPEC (vincristine, prednisolone, etoposide and chlorambucil) in a single sequence of treatments without a gap because there was uncertainty about the completeness of the remission on ChlVPP alone. Only one patient whose disease clearly did not enter complete remission on ChlVPP (patient 7 in Table VI) appears to have been cured by the HOPE-Bleo regimen. No patients who had received previous adriamycin chemotherapy achieved lasting complete remission.

Among the 11 patients who had never previously entered complete remission, six entered complete remission on HOPE-Bleo (55%) but five of these relapsed after only 5–8 months of complete remission, the remaining patient (no. 7 in Table VI) has obtained a durable CR.

Curves for survival and durability of complete remission are shown in Figures 1 and 2 for all patients.

**Figure 1** Survival for all patients treated with HOPE-Bleo.

**Figure 2** Freedom from relapse for 26 patients achieving complete remission on HOPE-Bleo.

*Toxicity*

Table VII summarises the toxicity from HOPE-Bleo chemotherapy according to WHO grades (WHO, 1979); for each patient the maximum toxicity experienced at any time during treatment is shown. The principal toxicity was myelosuppression and 23% of patients suffered grade 3–4 leucopenia at some point during treatment. There were two toxic deaths. One due to neutropenic sepsis at day 13 and the other due to acute peritonitis without a microbiological diagnosis or apparent perforated viscus occurring at day 17. Although the explanation for the acute abdomen remains unclear we have regarded it as a toxic complication of the treatment.

The number of patients receiving each course of chemotherapy, together with course by course details of treatment delays and the number of drugs requiring dose reduction, is shown in Table VIII. Delays in chemotherapy administration were necessary in about 20% of patients. Dose reduction of etoposide was necessary in 5–8 patients during each of the first six courses according to nadir blood counts.

**Table VII** Toxicity

| | *Percentage of patients*[a] *in each WHO grade* | | | | |
|---|---|---|---|---|---|
| | *None* | *1* | *2* | *3* | *4* |
| Anaemia | 57 | 20 | 14 | 7 | 2 |
| Leucopenia | 36 | 16 | 25 | 7 | 16 |
| Thrombocytopenia | 91 | 0 | 5 | 0 | 5 |
| Nausea and vomiting | 41 | 30 | 23 | 7 | 0 |
| Alopecia | 30 | 5 | 16 | 48 | 2 |
| Neuropathy | 55 | 23 | 18 | 5 | 0 |
| Infection | 59 | 7 | 9 | 20 | 2 |
| Diarrhoea | 93 | 2 | 2 | 2 | 0 |
| Stomatitis | 84 | 7 | 7 | 2 | 0 |
| Other recorded | 95 | 0 | 5 | 2 | 0 |

[a]Data complete on all 44 patients. For each toxicity the worst toxicity experienced by each patient at any time during treatment is shown.

**Table VIII** Drug reduction and delay by course of treatment

| | Number of course | | | | | | | |
|---|---|---|---|---|---|---|---|---|
| | *1* | *2* | *3* | *4* | *5* | *6* | *7* | *8* |
| Number of patients | 44 | 41 | 39 | 33 | 30 | 24 | 3 | 2 |
| Number of patients with reduction of one or more drugs | | | | | | | | |
| Any reduction | 9 | 13 | 15 | 14 | 13 | 8 | 1 | 1 |
| 1 drug reduced | 5 | 8 | 8 | 7 | 6 | 5 | 1 | 1 |
| 2 drugs reduced | 2 | 3 | 5 | 4 | 4 | 1 | 0 | 0 |
| 3 drugs reduced | 1 | 1 | 1 | 2 | 2 | 1 | 0 | 0 |
| 4 drugs reduced | 1 | 1 | 1 | 1 | 1 | 1 | 0 | 0 |
| Number of patients with treatment delay | | | | | | | | |
| Any delay | – | 9 | 8 | 9 | 9 | 6 | 1 | 1 |
| 1 week delay | – | 6 | 7 | 9 | 8 | 5 | 1 | 0 |
| >1 week delay | – | 3 | 1 | 0 | 1 | 1 | 0 | 1 |

### Discussion

The HOPE-Bleo regimen is an effective treatment for relapsed or resistant Hodgkin's disease. It is readily delivered in the outpatient clinic provided that careful attention is given to haematological monitoring, particularly at the nadir following each course of treatment as myelosuppression was severe in a small proportion of these pretreated patients. Acute subjective toxicity was moderate with alopecia and mild gastrointestinal toxicity occuring in the majority of patients.

Comparison with existing reports is always difficult because of the differing patient populations studied. However, the majority of patients in this series had extranodal disease and B symptoms, the CR rate achieved suggests that the activity of HOPE-Bleo is probably as great as that of the ABVD regimen with a similar number of prolonged remissions (see Canellos *et al.*, 1987 for review). As expected the complete remission rate and the probability of a long term remission were both related to the previous sensitivity of the patient's disease to treatment and to the amount of previous chemotherapy received, as judged by the number of regimens and the previous use of drugs other than those included in the basic MOPP regimen or its variants. These data and the available literature do not, however, allow us to conclude with certainty that second line treatment with adriamycin containing combinations is superior to retreatment with MOPP-based regimens for patients relapsing from a long MOPP-induced CR (Fisher *et al.*, 1979; Canellos *et al.*, 1987).

These data with long follow-up in a moderate number of relapsed patients suggest that HOPE-Bleo might prove to be as effective as the ABVD regimen as a primary treatment for Hodgkin's disease, and in view of its ease of administration, modest acute toxicity and low probabilty of carcinogenesis or infertility we feel that this possibility should be further explored. The results of studies where MOPP or related regimens are alternated with etoposide containing regimens similar to HOPE-Bleo are awaited with interest and preliminary results are encouraging (M. Cullen and B. Hancock, personal communications).

Some lessons can be inferred from the results of this study for the management of Hodgkin's disease in relapse. Overall only a small proportion of patients who relapse following chemotherapy are likely to be cured by a subsequent combination chemotherapy regimen. Nevertheless, eight patients in this study, who had had only one or two previous treatment regimens, have achieved long disease-free intervals and are probably cured. The most powerful factors predicting for a good prognosis are, in combination, a small amount of previous chemotherapy and a long previous remission, and among 19 patients, in this study, who had relapsed from complete remission achieved by a single previous chemotherapy regimen, six (32%) achieved long-term complete remission.

These results suggest caution in the use of intensive and dangerous consolidation treatment such as high dose chemotherapy with autologous bone marrow transplantation (Zulian *et al.*, 1989; Russell *et al.*, 1989; Canellos *et al.*, 1987). Patients who enter CR on initial chemotherapy, relapse and then re-enter CR on second line chemotherapy have about a 30% chance of achieving long remission or cure and for such patients a policy of observation, perhaps with marrow cryopreservation, may be advisable. If, however, a patient fails to enter CR or has relapsed from CR more than once, and has B symptoms and extra nodal disease then the prognosis is very poor and the place of intensive consolidation should be considered. Such policies can now be tested in randomised prospective trials.

### References

BONADONNA, G., SANTORO, A., VALAGUSSA, P. & 4 others (1985). Current status of the Milan trials of Hodgkin's disease in adults. In *Malignant Lymphomas and Hodgkin's Disease*, Cavalli, F., Bonadonna, G. & Rejencweig, K. (eds) p. 299. Martinus Nijhoff: The Hague.

CANELLOS, G., SELBY, P. & MCELWAIN, T.J. (1987). Chemotherapy of Hodgkin's disease (II). In *Hodgkin's Disease*, Selby, P. & McElwain T.J. (eds) p. 285. Blackwell Scientific Publications, Oxford, London and Boston.

CARBONE, P.P., KAPLAN, H.S., MUSSHOFF, K., SMITHERS, D.W. & TUBIANA, M. (1971). Report of the Committee on Hodgkin's disease staging. *Cancer Res.*, **31**, 1860.

COLMAN, M. & SELBY, P. (1987). Second Malignancies and Hodgkin's disease. In *Hodgkin's Disease*, Selby, P. & McElwain, T.J. (eds) p. 361. Blackwell Scientific Publications: Oxford, London & Boston.

DE VITA, V.T., SIMON, R.M., HUBBARD, S.M. & 6 others (1980). Curability of advanced Hodgkin's disease with chemotherapy. *Ann. Intern. Med.*, **92**, 1949.

FISHER, R.I., DE VITA, V.T., HUBBARD, S.P., SIMON, R. & YOUNG, R.C. (1979). Prolonged disease-free survival in Hodgkin's disease with MOPP reinduction after first relapse. *Ann. Intern. Med.*, **90**, 761.

KAPLAN, E.L. & MEIER, P. (1958). Non-parametric estimation from incomplete observations. *J. Am. Stat. Assoc.*, **53**, 457.

LUKES, R.J. & BUTLER, J.J. (1966). The pathology and nomenclature of Hodgkin's disease. *Cancer Res.*, **26**, 1063.

RUSSELL, J.A., SELBY, P.J., REUTLER, B.A. & 7 others (1989). Treatment of advanced Hodgkin's disease with high dose melphalan and autologous bone marrow transplantation. *Bone Marrow Transplant.*, **4**, 425.

SANTORO, A., BONFANTE, V. & BONADONNA, G. (1982). Salvage chemotherapy with ABVD in MOPP-resistant Hodgkin's disease. *Ann. Intern. Med.*, **96**, 139.

SELBY, P., MCELWAIN, T.J. & CANELLOS, G. (1987). Chemotherapy of Hodgkin's disease (I). In *Hodgkin's Disease*, Selby, P. & McElwain, T.J. (eds) p. 269. Blackwell Scientific Publications: Oxford, London and Boston.

SELBY, P.J., PATEL, P., MILAN, S. & 8 others (1990). Ch1VPP combination chemotherapy for Hodgkin's disease: long-term results. *Br. J. Cancer* (in the press).

SUTCLIFFE, S.B. (1987). Infertility and gonadal function in Hodgkin's disease. In *Hodgkin's Disease*, Selby, P. & McElwain, T.J. (eds) p. 339. Blackwell Scientific Publications: Oxford, London and Boston.

TAYLOR, R.E., MCELWAIN, T.J., BARRETT, A. & PECKHAM, M.J. (1982). Etoposide as a single agent in relapsed advanced lymphomas. *Cancer Chemother. Pharmacol.*, **7**, 175.

WORLD HEALTH ORGANIZATION (1979). *WHO Handbook for Reporting Results of Cancer Treatment*. Offset Publication no. 48. WHO: Geneva.

ZULIAN, G.B., SELBY, P., MILAN, S. & 5 others (1989). High dose melphalan, BCNU, and etoposide with autologous bone marrow transplantation for Hodgkin's disease. *Br. J. Cancer*, **59**, 631.